# Effects of Microfiltration Pretreatment and Feed pH Adjustment on Nanofiltration and Reverse Osmosis Desalination of Agricultural Drainage Water

**DOI:** 10.3390/membranes16100310

**Published:** 2026-09-23

**Authors:** Ertuğrul Gül

**Affiliations:** Department of Environmental Health, Vocational School of Health Services, Hakkari University, 30000 Hakkari, Türkiye; ertugrulgul@hakkari.edu.tr

**Keywords:** agricultural drainage water, reverse osmosis, nanofiltration, microfiltration pretreatment, pH adjustment, membrane fouling, brackish water, Harran Plain

## Abstract

Agricultural drainage water is a saline return flow whose reuse is constrained in intensively cultivated basins such as the Harran Plain in southeastern Türkiye. This exploratory, descriptive bench study examined microfiltration (MF) pretreatment and adjustment of the feed pH to approximately 7 on real drainage water (4.8 to 8.1 mS cm^−1^) with flat-sheet NF270 and XLE membranes at 8 bar. Six treatment trains, four forming a 2 × 2 factorial, were each run twice in 6 h batch concentration mode, with the two runs of each train made on separate days. XLE rejected 93 to 98% of feed conductivity and delivered 0.10 to 0.63 mS cm^−1^ permeate, whereas NF270 rejected 76 to 80%. The second run of every factorial cell rejected more than the first, so run order was blocked post hoc. The blocked model associates MF pretreatment with a gain of 2.0 percentage points (95% CI 0.03 to 3.97) and bounds the pH effect within two points of zero. No result reaches the 0.05 level under familywise adjustment. Normalizing flux to net driving pressure separates osmotic loss from loss of membrane permeability; one unpretreated run of eight lost permeability, an observation compatible with chance. Water recovery was not measured, and irrigation suitability was not assessed, as conductivity alone cannot establish it. These runs tested acute rejection and flux, not the scaling control that motivates acid dosing in practice; the ions that set scaling potential were not measured. The findings are preliminary and describe process configuration of these feeds rather than membrane-level mechanisms.

## 1. Introduction

Artificial drainage of irrigated land is standard practice in semi-arid agricultural regions, where it prevents waterlogging and the accumulation of salts in the crop root zone [1]. The drainage water it produces is voluminous and saline: in Egypt alone, 12 to 14 billion m^3^ are generated annually, an amount that creates a considerable opportunity for water recovery if the salinity can be removed [2]. In the San Joaquin Valley, tile drainage water carries 3000 to 15,000 mg L^−1^ of total dissolved solids against an acceptable irrigation limit of roughly 750 mg L^−1^, so reuse necessarily implies desalination [1]. Drainage water also carries suspended and colloidal matter, residual agrochemicals, and a diverse microbial population [1,2], so affordable pretreatment is as much a design question as the desalination step. Agricultural drainage waters reported elsewhere span total dissolved solids of roughly 3000 to 30,000 mg L^−1^ [3,4]. Achievable recovery on such waters is commonly limited by saturation with respect to gypsum, calcite, and silica rather than the membrane itself [3,5].

The Harran Plain of Şanlıurfa Province in southeastern Türkiye combines all of these conditions. The plain covers about 152,000 ha of irrigable land developed under the Southeastern Anatolia Project in a semi-arid setting that receives 300 mm of rain a year against evaporation reaching 2023 mm [6]. Irrigation with good water, 0.38 dS m^−1^ from the Atatürk Dam, has been accompanied by a rising and saline water table. At a non-irrigated site near Akçakale, the salinity of the upper 20 cm of soil climbs from 10.9 dS m^−1^ in February to 28.3 dS m^−1^ in November within a single year [6]. That is far above the tolerance of the crops grown there, which Çullu et al. [6] give as 1.7 dS m^−1^ for corn, 6.0 for wheat, and 7.7 for cotton. The problem is also spatially uneven: across 140 locations sampled after the crop season, soil conductivity in the top 30 cm ranged from 0.2 to 39.7 dS m^−1^ [7]. The shallow aquifer also carries the nitrate signature of intensive fertilizer use, with well concentrations reaching 550 mg L^−1^ [8], against 52.9 to 368 mg L^−1^ total dissolved solids in the fresh karst groundwater of the neighboring Balıklıgöl basin [9]. The main drainage channel of the plain discharges 190 to 200 million m^3^ per year, a volume that exceeds the annual drinking water demand of the province [10]. Any reuse of that water has to address the salt that irrigation mobilizes before the nutrients applied to it become relevant.

Among the membrane processes available for desalination, reverse osmosis (RO) and nanofiltration (NF) differ in the trade-off they offer between salt rejection and productivity. On brackish water, NF270 delivered 90.91 L m^−2^ h^−1^ at 10 bar against 41.38 L m^−2^ h^−1^ for the tighter NF90. At 60% recovery, the NF270 permeate still carried 1245 mg L^−1^ of dissolved solids from a feed of 2287.5 mg L^−1^. A reverse osmosis stage placed on that permeate rejected up to 97% of what reached it [11]. NF alone cannot bring high-salinity water down to potable specifications, although it performs well as a partial desalination or pre-RO step [12,13]. For brackish feeds in the 1000 to 10,000 mg L^−1^ range, RO is typically operated at 6 to 30 bar, far below the 60 to 80 bar of seawater systems [12], and low-pressure RO membranes can achieve high salt rejection with useful flux at such pressures [1]. In a direct comparison on the same feed, RO removed about 99.4% of dissolved metals against 97.4% for NF [14].

At a fixed membrane and pressure, two interventions remain available to an operator, and both are in common use. The first is pretreatment. Agenson and Urase [15] argue that preventing fouling upstream is preferable to cleaning after it and list microfiltration and ultrafiltration among the options for NF, in keeping with the general use of MF as a pretreatment step for RO [16]. Capillary membrane pretreatment produces RO feed water with a much lower concentration of colloidal and suspended particles than conventional multi-step pretreatment, and in municipal reuse service it reduces the eventual flux decline of an ESPA element from 85% to 60% [17]. Ultrafiltration membranes fabricated from natural minerals such as zeolite, smectite, and kaolin have been reported at production costs of USD 15 to 18 m^−2^ against 500 to 1000 USD m^−2^ for conventional ceramic membranes [18,19]. Such materials may widen the choice of low-cost pretreatment media. Fouling also reduces rejection. Organic fouling of a tight RO membrane lowered chloride rejection from 98.1% to 73.7% while cutting flux by 60%, whereas a loose NF membrane lost only 26% of its flux and slightly increased its chloride rejection [15]. The second intervention is feed pH adjustment. The pH of the solution governs the ionization of dissolved species and hence their rejection, with more strongly charged species rejected more effectively [20], and the polyamide skin layer itself carries a pH-dependent charge, being negatively charged above about pH 5 through dissociation of surface carboxyl groups [21]. A pH-dependent surface charge governs rejection on other membrane chemistries as well; on a ceramic ultrafiltration membrane, chromium(VI) rejection rose from 59% at pH 8 to 87% at pH 2 as protonation turned the surface charge positive [19]. That polyamide layer is formed by interfacial polymerization and is liable to hydrolysis in strongly acidic or alkaline media, for which pH-resistant chemistries such as polyurea are being developed [22]. The pH range examined here lies well inside of the window of a conventional polyamide membrane.

How these two interventions behave together on real agricultural drainage water at a fixed, modest operating pressure is less clear. Much of the evidence for pH effects comes from synthetic feed solutions rather than real waters [20], and pretreatment studies typically report flux protection without testing whether the same benefit appears on a looser NF membrane treating the same water. Scopus and Web of Science searches on membrane treatment of agricultural drainage water returned no study that compared an acidified feed against an unadjusted control on a real water. The practice is carried over from scaling control in continuous plants.

Recent work on drainage water separates the same two questions. The closest study to the present one is that of Pak et al. [23], who ran NF270, the tighter NF90, and a brackish RO membrane on a synthetic drainage water and reported conductivity rejections of 22 to 23% for NF270 in dead-end tests and 60% in cross-flow against 94% and about 97% for NF90 and the RO membrane under cross-flow. No pretreatment was applied ahead of the membranes, and pH was not a variable; their second axis was hydrodynamic, dead-end against cross-flow. Other studies that use real drainage water treat pretreatment as a given rather than as a factor. Hejase et al. [24] place a bioreactor and ultrafiltration ahead of the membranes; El Sayed et al. [25] raise the pH to 9.8 with sodium hydroxide inside of the pretreatment chemistry rather than at the membrane. Guy et al. [26] filter the reverse osmosis feed through 20 and 5 µm cartridges and dose acid to hold the electrodialysis diluate near pH 7; and Dos Santos et al. [27] characterize the water without treating it. Membrane work on this particular water has addressed its nutrient load rather than its salinity. Kayaalp [10] treated Harran Plain drainage water in a venturi-integrated anoxic membrane bioreactor and reported nitrate nitrogen removal above 90%, but the process was directed at the nitrogen load rather than salinity, which limits reuse. A direct comparison is therefore lacking in which both interventions are applied to the same water on the same rig at one documented pressure and their effects are measured against each other.

This study therefore evaluates microfiltration pretreatment and feed pH adjustment factorially on real Harran Plain drainage water. Four RO trains form a complete 2 × 2 factorial crossing MF pretreatment with pH adjustment on a low-energy XLE membrane, and two further trains give the MF factor on an NF270 membrane, all at a documented 8 bar and with permeate mass logged every 15 s over 6 h batch concentration runs. Two hypotheses are tested. The first is that microfiltration pretreatment raises the conductivity rejection of the reverse osmosis membrane; the second is that adjusting the feed pH to approximately 7 leaves rejection unchanged over these 6 h runs. Three objectives follow: to quantify the effect of each intervention on conductivity rejection and on flux behaviour, to establish what each of them can and cannot be shown to do on this water, and to report the resulting performance envelope for reuse. Part of this material was reported earlier in the final report of the project that produced it [28], and Section 2.1 sets out what is reused here and what is new.

## 2. Materials and Methods

### 2.1. Feed Water and Study Area

Agricultural drainage water was collected from open drainage discharge channels of the Harran Plain irrigation district near Akçakale, Şanlıurfa Province, Türkiye, during the irrigation seasons, between June and August, of two consecutive years. Sampling points were selected on channels known to carry saline return flow. The water was transported to the laboratory and passed through a coarse cartridge filter (CART) before entering the feed tank of the membrane system in all experiments. The initial electrical conductivity (EC) of the feed ranged from 4.8 to 8.1 mS cm^−1^ and the unadjusted feed pH from 7.9 to 8.3; per-run values are given with the experimental design in Section 2.3. In the laboratory, the water was held in a closed polyethylene storage tank at 20 °C. The storage tank was replenished with fresh collections before it ran empty, so the composition of the stored water changed over the series of runs, and the replenishments were not dated.

The drainage system supplying this water was characterized separately within the same project. Kayaalp [10] reports the aggregate statistics of that survey, which sampled seventeen locations on five occasions, with conductivity between 0.65 and 7.13 mS cm^−1^, pH between 7.1 and 8.4, nitrate nitrogen between 2.6 and 44.7 mg L^−1^, sulfate between 80 and 1200 mg L^−1^, phosphate phosphorus up to 1.4 mg L^−1^, and chemical oxygen demand between 2.5 and 47.8 mg L^−1^. The feeds of the present runs sit at and above the upper end of that conductivity range, which places their origin among the more saline parts of the network. Two grab sulfate measurements taken on the feed itself, 1250 and 1050 mg L^−1^ in the MF-pretreated NF and RO feeds, fall at and slightly above the top of the surveyed sulfate range and are consistent with the same attribution.

That characterization, however, describes the drainage network rather than the water used here. The sampling record gives the channels and the months of sampling, and, apart from the two sulfate measurements, no ion analysis was performed on the stored water itself. The ion data therefore describe the water body rather than the feed of the twelve runs, within which feed and permeate were followed by conductivity and pH only. Conductivity is accordingly used throughout as a bulk salinity proxy, and no ion-resolved rejection is reported apart from two grab sulfate pairs (Section 4). Calcium, magnesium, bicarbonate, chloride, sodium, and boron were not measured on any sample, so the ions that govern scaling risk are not available for this water. Alkalinity, hardness, total dissolved solids, turbidity, suspended solids, organic carbon, silt density index, and particle size were likewise not determined on the experimental feed, nor was nitrate.

These runs belong to the same project, TÜBİTAK 114Y453. Its final report [28] presented the first of the two runs of each train as tables of feed and permeate conductivity and pH at 30 min intervals together with the raw flux curves. Its conclusions rest on those single runs, reported without a measure of variability, flux normalization, or a statistical model. The present analysis returns to the same laboratory records. It adds the second run of every train, the permeate mass logged every 15 s, a flux referring to net driving pressure, and a factorial estimate with an equivalence test and a post hoc run-order block. Section 4 sets out where the earlier reading of the flux data and the present one diverge. No figure or table of the report is reproduced here; every figure and table in this manuscript is derived anew from the raw records. Two further outputs of the same project addressed other questions on this water: its nitrogen load [10] and the soil response to irrigation with it [29].

### 2.2. Membranes and Filtration System

Experiments used flat-sheet polyamide thin-film composite membranes. NF270 (Dow FilmTec, Minneapolis, MN, USA) rejects organic solutes above 400 Da and 40 to 60% of monovalent ions and gives 99.2% MgSO_4_ rejection at 2000 mg L^−1^ and 8.96 bar [30]. XLE (Dow FilmTec), an extra-low-energy brackish reverse osmosis membrane, is specified at a stabilized NaCl rejection of 99.0%, with a minimum of 97.0%, at 2000 mg L^−1^, 8.6 bar, and 15% recovery [31]. Both are manufacturer values at 25 °C; the XLE figures are element specifications, as no flat-sheet data sheet is published for that membrane. Microfiltration pretreatment used a SINAP-10 PVDF module (Shanghai SINAP Membrane Tech Co., Ltd., Shanghai, China; 0.1 µm nominal pore size, 0.1 m^2^ membrane area, 220 × 320 × 6 mm) operated at 0.5 bar. Its operating mode, the volume treated for each run, its flux and transmembrane pressure, and any backwashing or cleaning between runs were not recorded. The pretreated water was collected and held until the NF or RO membrane had been installed, and the run was then fed from it, including any water added to the feed tank during the run (Section 2.4). No before and after measurement was taken across the microfiltration step, so what the module removed from the feed cannot be stated.

The NF and RO experiments were run on a commercially assembled laboratory cross-flow skid built around a plate-and-frame membrane cell with an effective membrane area of 126 cm^2^ (0.0126 m^2^). The skid further comprised a feed tank, a high-pressure pump with a frequency converter, pressure gauges on the module and concentrate line, a concentrate rotameter, and a chiller. Membrane coupons were mounted with the active layer facing the feed channel, and the cell was closed with a hydraulic clamping pump at a clamping pressure of 60 to 70 bar. The applied transmembrane pressure was set to 8 bar on the module gauge for both NF and RO runs, as specified in the laboratory operating instructions. The permeate was collected at atmospheric pressure, so the transmembrane pressure is the module gauge reading itself, and no separate pressure drop term enters Equation (2). The concentrate flow was held at 3 L min^−1^ by the frequency converter, and feed temperature was controlled by the chiller set to 20 °C. The layout is shown in Figure 1, in which the 80 bar and 10 bar markings denote gauge ranges rather than operating pressures. The 80 bar gauge monitors the hydraulic clamping circuit, whereas the 10 bar gauge monitors the process line, which was operated at 8 bar with the safety valve set above 10 bar. The make, range, and calibration of the gauges were not recorded, and the inlet and retentate pressures were not measured separately, so the applied pressure represents the instructed module gauge setting rather than an independently validated value. The cell housing model, channel geometry and spacer type, cross-flow velocity, and pump identity are likewise undocumented.

### 2.3. Experimental Design

Six treatment trains (plans) were tested (Table 1). Plans 1 and 2 compare NF270 without and with MF pretreatment. Plans 3 to 6 form a complete 2 × 2 factorial on the XLE RO membrane, crossing MF pretreatment (without/with) with feed pH adjustment (unadjusted/adjusted to approximately 7 with dilute HCl). The pH factor was applied on the reverse osmosis membrane only. Crossing it with the NF270 arm as well would have required two further trains and four more runs that the campaign did not include, and the design record does not state why it was restricted in this way. For these results, the question is in any case chiefly one for the reverse osmosis membrane, as the NF270 permeate remained several times more saline than the reverse osmosis permeate throughout (Section 4). Each train was run twice, giving twelve drainage water runs, and pure water fluxes were measured in independent baseline runs (two NF, three RO).

The two runs of a train were performed on separate days, each drawing feed from the storage tank and using a coupon prepared as described below. They are therefore independent runs rather than repeated measurements of a single run, and the spread between them carries the difference in the feed drawn on the two occasions as well as run-to-run variability. Because the feed drawn for a run is nested within the run rather than crossed with the two factors, the design cannot fully separate a treatment effect from a feed effect; this bears on the flux results in particular and is taken up in Section 4. The initial conductivities differed between the two runs of a train by 7% or less in four trains but by 16% in Plan 2 and 30% in Plan 6 (Table 2). The MF-pretreated feeds also started with 19 and 28% more saline on average than the unpretreated ones on NF270 and XLE. Microfiltration does not retain dissolved salts, so this difference is not a property of the pretreatment. It may reflect the state of the storage tank when each run was drawn, as the replenishments came from different points of the drainage network whose conductivity varies widely (Section 2.1), or residual concentrate left in the rig, which the pretreated water entered twice, once for microfiltration and once for the NF or RO run. The record does not distinguish the two. The association between pretreatment and rejection therefore carries this imbalance, and the covariate analysis of Section 3.5 cannot remove it. Runs are therefore reported individually throughout, and the between-run spread within a treatment cell is used as the error term against which factor effects are judged (Section 2.5).

EC_f,0_ and pH_f,0_ are the feed conductivity and pH at the start of a run, EC_f,360_ and pH_f,360_ the same quantities at 360 min, and CF the ratio of the two conductivities. The recovery bound is inferred from CF on a closed salt balance rather than measured. It is given for the reverse osmosis runs alone, because the nanofiltration membranes passed too large a fraction of the salt for that balance to hold (n.a.). Every run was sampled at 30 min intervals, which gives 13 observations. All twelve runs were operated at 8 bar with the chiller set to 20 °C, but the feed temperature itself was not logged. The conductivities are referred to the reference temperature of the meter by its automatic compensation (Section 2.4), whereas the fluxes are not temperature-normalized. No measurement uncertainty is quoted beyond the manufacturer specifications of the meters given in Section 2.4 because no calibration record was kept.

### 2.4. Operating Procedure and Measurements

The campaign began with unused coupons, which were conditioned with pure water for at least 2 h because the manufacturer states that flux is not stable over this initial period; the pure water flux was read thereafter. Conditioning followed that instruction rather than a numerical stability criterion, and no such criterion was set in advance; the residual drift is quantified in Section 3.1. Pure water flux was recorded on five baseline runs, two on NF270 and three on XLE, and not on every coupon that went on to treat drainage water, so the baselines characterize the two membranes rather than each experimental run. After every run, the coupon was removed, cleaned through the cleaning procedure specified by the membrane manufacturer, and stored in pure water or in 1 g L^−1^ sodium metabisulfite solution until its next use. Every run after the first use of a coupon therefore began with a chemically cleaned rather than a new membrane. The chronological order of runs was not recorded, so the number of cleaning cycles behind any given coupon cannot be reconstructed, and coupon lot numbers and supplier batches were also not documented; no allocation or randomization procedure was recorded either. No antiscalant and no biocide were dosed in any run. The drainage water is naturally alkaline, at pH 7.9 to 8.3, and Plans 5 and 6 ask whether its removal changes when the feed is brought to neutral. The feed, including any water added during the run, was adjusted to approximately pH 7 with dilute hydrochloric acid. The concentration and dose were not recorded. No lower set point was included, as the question was confined to the removal of the natural alkalinity rather than acidic operation. Across the four adjusted runs, the feed stayed between pH 6.89 and 7.34 at all 13 sampling points and ended on average 0.01 units below where it started. All eight unadjusted runs rose instead by 0.25 to 0.69 units and by 0.47 on average (Table 2). The feed pH values of approximately 7 and 8 lie well within the operating window of commercial polyamide composite membranes, so the adjustment probed a separation response rather than membrane stability. Tolerance of strongly acidic or alkaline feeds is a separate membrane materials question pursued through pH-resistant chemistries such as polyurea hollow fiber membranes [22] and is not addressed by the present design.

Each run lasted 6 h in batch concentration mode: permeate was withdrawn continuously while the concentrate was recycled to the feed tank, so the feed concentrated over the run. The feed EC consequently rose by a factor of 1.3 to 2.7 depending on the run (Table 2), which allows rejection to be observed against rising feed salinity. When the level in the feed tank fell during a run, water was added to it from the storage tank or from the pretreated volume in the MF runs. The additions were not planned in advance, and their timing and volume were not recorded; they appear as a single-step decrease in feed conductivity in seven of the twelve runs (Section 3.4). The feed tank volume and the added volumes were not recorded, so water recovery was not measured. It can, however, be bounded from the concentration factor, because the concentrate was recycled and the salt fed to the tank in the initial charge and in the additions stayed in the retentate apart from what passed to the permeate. Recovery here is the permeate volume as a fraction of all water fed to the tank. Over the eight RO runs, the concentration factor ranged from 1.31 to 2.12, which corresponds to a recovery of roughly 25 to 54%. This range is a bound rather than a measurement, and it assumes that the water added during a run had the conductivity of the initial feed, that conductivity tracked the dissolved salt proportionally, and that evaporation and sampling losses were negligible. Permeate mass was collected on an A&D precision balance (A&D Company, Tokyo, Japan) and logged automatically every 15 s to 0.1 g (RsCOM software of the A&D WinCT package), which gave approximately 1400 readings per run. Because feed temperature was not logged during the runs and only the chiller setpoint is known, the reported fluxes are not temperature-normalized. Feed and permeate EC and pH were measured every 30 min at 13 sampling points per run. The pH meter was a WTW pH 3110 (WTW GmbH, Weilheim, Germany) with a SenTix 41 electrode, and the conductivity meter a WTW Cond 3110 with a TetraCon 325 four-electrode cell (cell constant 0.475 cm^−1^). The manufacturer specifies ±0.01 pH for the pH meter [32] and ±0.5% of the reading for conductivity, with automatic non-linear temperature compensation to the reference temperature [33]. Both meters were calibrated before use; the calibration records and the reference temperature setting were not kept.

(1) $$R=\left(\frac{{EC}_{f}-{EC}_{p}}{{EC}_{f}}\right)\times 100$$

where EC_f_ and EC_p_ are the feed and permeate conductivities at the same sampling time. Permeate flux J (L m^−2^ h^−1^) was calculated from the logged cumulative permeate volume over the effective membrane area. After each run, membranes were cleaned using the procedure specified by the manufacturer and stored in deionized water or 1 g L^−1^ sodium metabisulfite; the system was flushed without a membrane for 30 to 60 min between runs.

### 2.5. Data Processing

The raw balance logs contain occasional restarts (balance re-zeroed mid-run). Logs were therefore segmented at any cumulative volume drop or time discontinuity, and flux was computed within continuous segments only as the slope of a linear regression of cumulative volume in ±15 min windows on a 5 min grid. In several runs, the balance continued to log after permeate collection had stopped, leaving a flat tail of up to 15 min that a regression window reaching into it would report as a collapsing flux; trailing points whose local collection rate had fallen below 35% of the run’s typical rate were therefore removed before flux was computed. Reported point fluxes J_30_, J_120_, and J_240_ refer to elapsed run time; the time-averaged flux over 60 to 240 min is used for factor comparisons because it is available for all runs. Rejection was recomputed from the raw feed and permeate conductivities. Balance restarts or time discontinuities occurred in eight of the twelve drainage water runs and in three of the five baselines, and every segment boundary and excluded tail is listed in the Supplementary Materials. The tails removed were 0.25 to 13.25 min long in six runs and absent in the other six. Lowering the threshold to 20% of the typical rate or raising it to 50% leaves the 60 to 240 min window mean of every run unchanged to two decimals because that window never reaches the tail.

Because the runs concentrate their own feed, a falling flux does not by itself indicate fouling: the osmotic pressure of the feed rises as the batch concentrates and the net driving pressure falls accordingly. Permeability was therefore computed as

(2) $$A=\frac{J}{\left(\Delta P-\Delta \pi \right)}$$

with ΔP the applied 8 bar and Δπ estimated from the measured feed and permeate conductivities. Because the conversion from conductivity to osmotic pressure is an estimate, the analysis was repeated over a grid of plausible values for the two conversion coefficients for a feed-side concentration polarization factor and for the applied pressure; scenarios implying a net driving pressure below 1.5 bar were discarded as incompatible with the observed fluxes. The three levels of each of the four assumptions are listed in Section 3.5, and the full grid of 81 scenarios, with the admissibility flag and the two effect estimates of each, is provided in the Supplementary Materials. One property of this correction should be kept in view when reading the permeability traces: because the net driving pressure appears in the denominator, A becomes progressively more sensitive to the estimate as the batch concentrates. In the runs that concentrated the most, the net driving pressure fell from about 5.4 to below 3 bar, and the apparent permeability of those runs rose by up to 60% over the run. That rise tracks the shrinking denominator, not any gain in the membrane, so the correction is used here to identify runs that lose permeability while their driving force is still largely intact, not to rank runs by permeability gain.

Factor effects on mean conductivity rejection were estimated over Plans 3 to 6 from five fits of the same eight runs; the fits differ only in how residual variation is modeled. In every fit, the response is the run mean rejection of Table 3; the two factors enter as indicator variables equal to 1 when the intervention was applied, the interaction is their product, and the run order block is an indicator for the second run of each cell; the coefficients of all five fits are collected in a table in Section 3.5. An additive model without blocking, in which rejection was regressed on microfiltration and pH, leaves 5 residual degrees of freedom with a residual standard deviation of 1.69 percentage points. The corresponding full factorial model, which adds their interaction, leaves 4 residual degrees of freedom with a residual standard deviation of 1.88 points. Because inspection showed the second run of every cell to be displaced in the same direction (Section 3.5), both models were also fitted with run order entered as a block. Blocking leaves 4 residual degrees of freedom (residual standard deviation 1.00 points) in the additive form and 3 residual degrees of freedom (1.15 points) in the full factorial form. The classical 2 × 2 estimator, which pools the within-cell variances of the four cells, has 4 residual degrees of freedom and a pooled standard deviation of 1.88 points. The design is balanced and orthogonal, so the point estimate of the microfiltration main effect, +2.00 percentage points, is identical in the two additive fits and in the pooled estimator, and only its standard error and the residual degrees of freedom change. In the two full factorial fits, the microfiltration coefficient is the simple effect at unadjusted pH, +1.83 points, and the main effect is recovered by adding half of the interaction. The blocked additive model is the primary analysis because the two runs of each treatment were separated in time and in the feed drawn for them. The remaining fits are reported to keep the sensitivity of the intervals to this choice visible. In its fullest form, the model is

(3) $$\bar{R}=\beta_{0}+\beta_{MF}\cdot MF+\beta_{pH}\cdot pH+\beta_{MF\times pH}\cdot MF\cdot pH+\gamma \cdot B+\varepsilon$$

where $\bar{R}$ is the run mean rejection, MF and pH are indicator variables equal to 1 when the intervention was applied, B is an indicator for the second run of a cell, and ε is the residual. The additive fits drop the interaction term, the unblocked fits drop B, and the pooled 2 × 2 estimator is the unblocked full factorial with the within-cell variances pooled as the error term.

The interaction of microfiltration with pH was estimated in the pooled estimator and in the blocked full factorial model; retention of the additive forms rests on those estimates (Section 3.5). A separate test of each factor against zero does not compare the two, so their difference was estimated as a contrast within the blocked additive model. A failure to reject zero is likewise not evidence of absence, so the pH effect was further assessed by two one-sided tests. For this procedure, the 90% confidence interval is the operative summary, and it is reported alongside the conventional 95% interval. No equivalence margin was specified in advance of the experiment; the margins examined were chosen after the data were seen, so the resulting exclusions are descriptive rather than a formal equivalence claim. Several tests are reported on the same eight runs, and no correction for multiplicity is applied, because these are descriptive summaries of one small experiment rather than a pre-specified confirmatory family. Holm and Bonferroni adjustments are nevertheless reported with the estimates, and none of the effects survives them at the 0.05 level (Section 3.5). Residual diagnostics beyond the collinearity check of Section 3.5 were not pursued, as 3 to 5 residual degrees of freedom leave them without power.

Initial feed conductivity was not balanced across the pretreatment arms, averaging 5.16 mS cm^−1^ in the cells without microfiltration and 6.57 mS cm^−1^ in those with it. The microfiltration effect was therefore re-estimated with feed conductivity as a covariate using the initial and the window mean values in turn, each on 4 residual degrees of freedom. A further fit carried the covariate and the block together on 3 residual degrees of freedom. The outcome of these adjusted fits, together with the collinearity between the pretreatment and the covariate, is reported with the main results (Section 3.5). A separate check removed the osmotic correction outright and re-estimated the same factor effects on the uncorrected flux and permeability values. Effects that were unresolved with the correction remained unresolved without it (Section 3.5). Rejection cannot be affected by this check, as it is computed from measured conductivities and does not pass through the osmotic correction. The analysis notebook, the derived time series, the summary tables, and the complete model outputs are provided as Supplementary Materials.

## 3. Results

### 3.1. Baseline Pure Water Fluxes and Operating Envelope

Pure water fluxes at 120 min were 98 and 126 L m^−2^ h^−1^ for the two NF270 baselines and 59, 65, and 67 L m^−2^ h^−1^ for the three XLE baselines. Permeability windows derived from the manufacturer specifications [30,31] are 15.1 to 20.5 L m^−2^ h^−1^ bar^−1^ for NF270 and 6.7 to 7.8 L m^−2^ h^−1^ bar^−1^ for XLE. Both windows are permeabilities referring to net driving pressure, the catalogue flux divided by the applied pressure less the osmotic pressure of the test feed, which leaves 8.1 bar for NF270 and 6.9 bar for XLE. DuPont publishes no flat-sheet data sheet for XLE, so its window is derived from the element specification. Dividing the measured fluxes by these windows implies an applied pressure of 4.8 to 8.3 bar for NF270 and 7.6 to 9.9 bar for XLE. Both ranges are consistent with the instructed 8 bar setting, but this consistency is not an independent validation of the setpoint. Coupon permeability and compaction vary between samples, and the same manufacturer figure enters both the expectation and the check, so the calculation can demonstrate consistency only. The pure water runs were themselves not fully stable. Over 240 min, one NF270 baseline lost 15.1% of its first-reading flux, whereas the other gained 1.9%. Two XLE baselines lost 7.9 and 12.5% over 240 min, and the third, which ended at 155 min, had lost 11.1% by then. Drift of this magnitude must be allowed for when the flux declines of the drainage water runs are interpreted. Coupon to coupon reproducibility also differed between the two membranes. Clean water permeabilities of the three XLE coupons, on which the factorial design rests, spread by 10.2% of the mean at 30 min, 11.3% at 120 min, and 1.6% at 240 min. Four of the eight XLE values fall inside of that window and four sit above it, the highest by 10%. A coupon is expected to read above an element figure because the element specification carries the hydraulic losses of a full-length pressure vessel, so the offset is not evidence against the 8 bar setting. The two NF270 coupons differed by 24.9% at 120 min and 21.2% at 240 min, and the permeability of the second, 12.26 and 12.50 L m^−2^ h^−1^ bar^−1^, fell entirely below the window. On coupon grounds alone, the NF arm is therefore the weaker of the two, whereas the XLE coupons agree closely enough to support the factorial comparison.

### 3.2. Permeate Flux Behaviour

Figure 2 collects the flux behaviour of all twelve drainage water runs, whose plan labels are defined in Table 1. Its upper row shows the raw permeate flux of the NF270 trains (Figure 2a) and of the XLE trains without (Figure 2b) and with MF pretreatment (Figure 2c), with the two runs of each train drawn separately. The lower row (Figure 2d–f) expresses the same runs as permeability relative to the start of the run, the correction developed in Section 2.5 and applied in Section 3.3.

On drainage water, NF fluxes at 30 min were 63 to 76 L m^−2^ h^−1^, roughly 40% below the pure water baseline, and fell to 56 to 64 L m^−2^ h^−1^ by 240 min, the four runs declining along near-parallel paths regardless of pretreatment (Figure 2a). Their time-averaged flux over 60 to 240 min was 67.0 and 67.6 L m^−2^ h^−1^ without MF and 70.1 and 58.9 L m^−2^ h^−1^ with it, so the two conditions are not separated; the spread between the two MF runs is wider than the distance between the treatment means.

RO fluxes were lower and more variable between runs (Figure 2b,c). The steepest decline of the whole study occurred in the first unpretreated run (Plan 3), where the flux fell from 49 to 27 L m^−2^ h^−1^ between 50 and 240 min, whereas its companion run was almost stable (29 to 32 L m^−2^ h^−1^). Both patterns are visible in Figure 2b, where the two runs of each train also start from clearly different levels, the difference in initial feed conductivity between the two occasions (Section 2.3). The MF-pretreated runs decline more gently, though from starting levels that are just as scattered (Figure 2c). The time-averaged flux over 60 to 240 min was 37.6 (Plan 3, runs 41.9 and 33.3), 38.4 (Plan 4, 40.9 and 35.9), 37.1 (Plan 5, 44.1 and 30.1), and 27.4 L m^−2^ h^−1^ (Plan 6, 24.6 and 30.2) (Table 3). Only the last of these sits below the others, and the two runs of a train differ by as much as 14 L m^−2^ h^−1^, so no flux difference can be read from the means alone. Section 3.3 separates what part of these declines is osmotic.

### 3.3. Separating the Osmotic and the Membrane Components of the Flux Decline

Because every run concentrates its own feed, part of the observed flux decline is simply the loss of driving force as the osmotic pressure of the feed rises. Expressed as permeability between 50 and 240 min, the flux of seven of the eight RO runs holds between 0.83 and 1.20 of its 50 min value, so their falling flux is accounted for by the falling net driving pressure alone (Figure 2e,f). The remaining run, on unpretreated feed as received, retains 0.57 at 240 min and 0.46 by the end of the run, and it does so while its net driving pressure falls only from 5.71 to 5.39 bar. That is a 6% loss of driving force against a 46% loss of flux, which rules out the osmotic explanation for that run (Figure 2e). It is also the run with the steepest raw decline. The ratio for that run stays between 0.56 and 0.67 across every parameterization of the correction examined in Section 2.5, so identification does not rest on the chosen coefficients. The upward drift of several other runs is an artefact of the same correction, as the net driving pressure enters the denominator and falls to below 3 bar in the runs that concentrate most (Figure 2e,f); those runs are not evidence of a membrane improving.

The shape of the decline bears on whether a mechanical fault could account for the anomalous run. Its flux was flat between 50 and 100 min (48.9 to 49.1 L m^−2^ h^−1^) and fell gradually to 38.7 L m^−2^ h^−1^ by 200 min. It then fell steeply to 23.0 L m^−2^ h^−1^ by 250 min and settled near 20 L m^−2^ h^−1^. An induction period followed by an accelerating decline is difficult to reconcile with a leaking gasket or a poorly seated cell, which would produce a step change. The two restarts of the balance log both fall within the first 31 min, 170 min before the decline begins. Over the comparable window, the clean water XLE baselines lost 7.9 to 12.5% of their permeability, whereas this run lost 43%. The shape and the size of the loss separate the run from compaction, instrument drift, and a mechanical fault. They cannot identify a deposit because no membrane autopsy, foulant analysis, or same coupon permeability measurement before and after cleaning was performed.

On NF270, the same treatment leaves nothing comparable: the four NF runs end between 0.89 and 1.31 of their 50 min permeability, so the loose membrane shows no loss of the kind seen in that one RO run (Figure 2d). The raw flux curves nonetheless overstate what the corrected data support. Over 6 h at this recovery, a genuine loss of membrane permeability was observed once, on unpretreated feed, and not at all in the four MF-pretreated runs (Figure 2e,f). One event in eight runs does not support an estimate of frequency: the corresponding contingency, one loss in four unpretreated runs against none in four pretreated ones, is entirely compatible with chance (Fisher exact test, *p* = 1.00). It is reported here because it is the only apparent permeability loss consistent with fouling in the study and because its timing and shape are informative, not as evidence that pretreatment prevents such losses.

### 3.4. Conductivity Rejection Under Batch Concentration

Figure 3, on the same plan labels, pairs the rejection trajectory of every run (Figure 3a–c) with the feed conductivity against which it was achieved (Figure 3d–f) in the same three columns as Figure 2. Feed EC rose over every run by a factor of 1.6 to 2.7 in the NF runs and 1.3 to 2.1 in the RO runs, with a single-step decrease at one sampling point in the seven runs in which water was added to the feed tank (Section 2.4, Figure 3d–f). NF270 rejected 76 to 80% of feed conductivity on average, and rejection rose over the course of each run, from 72 to 80% at the start to 79 to 85% at 360 min, even as the feed concentrated (Figure 3a); permeate EC stayed between 1.23 and 1.98 mS cm^−1^. On XLE, rejection separates by pretreatment rather than by pH branch; the four unpretreated runs spread over roughly 90 to 98%, with the first run of each train sitting below its second throughout (Figure 3b), whereas the four MF-pretreated runs hold a narrow band between 96 and 98% for most of the run (Figure 3c). Across all eight runs, XLE rejected 93 to 98% on average and delivered a permeate between 0.10 and 0.63 mS cm^−1^. The two available sulfate measurements are consistent with this picture, showing 88% removal in the MF and NF train (Plan 2) and 93% in the MF and RO train (Plan 4). Because the runs reached different concentration factors, rejection was also read at a matched concentration factor of 1.3, the largest value attained by every RO run. At that point, the unpretreated runs rejected 93.4 to 98.0% and the MF-pretreated runs 97.4 to 98.1%, so the difference between the cell means, 2.1 percentage points, matches the run mean contrast of Section 3.5. The runs at pH 7 rejected 93.4 to 98.0% against 94.4 to 98.1% for the feed as received. The volume-weighted permeate conductivity of each run, obtained by weighting the 30 min permeate readings with the permeate volume collected between them, lies within 0.05 mS cm^−1^ of the time mean and is given in Table 3.

### 3.5. Factorial Assessment of MF Pretreatment and pH Adjustment

Figure 4 lays out the factorial evidence. The eight RO runs appear against the two levels of pretreatment for the feed as received (Figure 4a) and at pH 7 (Figure 4b). Within a panel, each line joins the two rejections of one replicate, solid for the first run of a cell and dashed for the second. A rising line therefore reports the difference within one replicate pair rather than the difference between cell means. The estimated effects follow as points carrying horizontal bars for their 95% confidence intervals. Each treatment is given twice, with the run order block and in the paler tone without it, against a vertical line at no effect (Figure 4c). On the RO membrane, mean rejection was higher in the MF-pretreated trains in both pH branches at 97.5 against 95.6% without pH adjustment and 97.3 against 95.1% with it (Figure 4a,b). The MF trains span 97.0 to 98.0% and 96.9 to 97.6%, against 93.8 to 97.5% and 93.3 to 96.9% for the unpretreated trains, so the ranges overlap at their edges. Mean rejection differed by 0.5 percentage points or less between the feed as received and at pH 7 at either level of the MF factor. The flux difference between the two pH levels was confined to the MF-pretreated branch, where the mean fell from 38.4 to 27.4 L m^−2^ h^−1^, while in the unpretreated branch the means were indistinguishable (37.1 against 37.6 L m^−2^ h^−1^). On NF, mean rejection did not differ with pretreatment at 78.8% without MF and 77.8% with it. Per-run feed properties and performance are given in Table 2 and Table 3.

With the effects estimated from the additive model without blocking, on 5 residual degrees of freedom (Table 4), the MF contrast on rejection was +2.0 percentage points (95% CI −1.1 to +5.1) and the pH contrast was −0.4 points (−3.4 to +2.7); on flux, the estimates were −4.4 (−16.4 to +7.6) and −5.8 L m^−2^ h^−1^ (−17.7 to +6.2). On that analysis, no interval excludes zero.

That analysis, however, treats the difference between the two runs of a cell as random error, which the data do not support. In all four cells, the second run rejected more than the first by 3.69, 1.01, 3.64, and 0.71 percentage points, a mean shift of 2.26 points in a single direction. The four shifts span a five-fold range, so the block is common in direction rather than in size, and with one replicate pair per cell its interaction with either treatment cannot be tested. Four cells moving the same way would have probability 0.125 if the direction were random, and the shift is larger than either treatment effect. The two runs of a train were made on separate days (Section 2.3), so run order carries whatever differed between the two occasions in the feed drawn, coupon history, and rig condition, rather than replicate noise. Entering run order as a block gives a residual standard deviation of 1.00 percentage points against 1.69 without it and resolves the effects differently: the block itself accounts for +2.26 points (95% CI +0.29 to +4.23, *p* = 0.033), MF for +2.00 points (+0.03 to +3.97, *p* = 0.048), and pH −0.38 points (−2.34 to +1.59, *p* = 0.62) (Figure 4c). The block does not produce the microfiltration estimate. The design is balanced, so run order is orthogonal to both treatment contrasts, and the two treatment estimates are identical to numerical precision whether the block is fitted or not. What the block changes is the residual variance, and with it the width of every interval. The estimate is therefore not an artefact of the block, although its separation from zero is, because the unblocked interval reported above spans zero. On flux and on permeability, nothing is resolved with or without the block.

That flux and permeability conclusion does not depend on how the osmotic correction is parameterized. Figure 5 repeats the estimation of the two factor effects on permeability across the correction grid of Section 2.5. Each of the four assumptions took three levels. The conductivity to solids coefficient ran at 0.55, 0.64, and 0.75 mg L^−1^ per µS cm^−1^ and the solids to osmotic-pressure coefficient at 6, 7, and 8 × 10^−4^ bar per mg L^−1^. The concentration polarization factor took 1.00, 1.15, and 1.30 and the applied pressure 7, 8, and 9 bar. Of the 81 combinations, 66 satisfy the admissibility rule that no run may fall below a net driving pressure of 1.5 bar. The 15 that fail pair the largest conversion coefficients and polarization factor with the lowest assumed pressure. Every admissible scenario was refitted with the pooled 2 × 2 estimator on 4 degrees of freedom, and each point is one scenario, the short horizontal bar marking the median of the scenarios that share a level (Figure 5a). At the nominal setting, a polarization factor of 1.00 at 8 bar, the pH effect is −0.53 L m^−2^ h^−1^ bar^−1^. Across the grid, it moves between −0.73 and +0.37 and changes signs in 16 of the 66 scenarios, while the microfiltration effect stays between −0.25 and +1.75. Neither t statistic approaches the threshold of ±2.78 in any scenario, the largest values reaching 0.95 for pretreatment and 0.90 for pH (Figure 5b).

Two qualifications bound this result. The two interventions are resolved differently against zero, but they are not resolved from each other; the contrast between them is 2.38 percentage points with a 95% interval from −0.41 to +5.16 (*p* = 0.077), so the data do not establish that pretreatment outperforms acid dosing, only that one effect clears zero and the other does not. The pH result is also more than a failure to reject. Two one-sided tests place the pH effect within ±2 percentage points of zero (*p* = 0.042) and within ±3 points (*p* = 0.010), while an effect as small as ±1 points cannot be excluded (*p* = 0.21). The 90% confidence interval underlying this procedure spans −1.89 to +1.13 percentage points. These margins were not specified in advance, so the exclusions are descriptive, and the result is a bound rather than a null. Adjusting the feed to pH 7 does not change conductivity rejection by more than about two percentage points in either direction.

The additive models behind these estimates were themselves examined, and every fit is set out in Table 4. The interaction of microfiltration with pH was estimated at +0.17 percentage points (95% CI −3.52 to +3.87, *p* = 0.90) by the pooled within-cell estimator. The blocked full factorial model gave +0.35 points (95% CI −4.82 to +5.51, *p* = 0.85) on 3 residual degrees of freedom. Both estimates are consistent with additivity, although 3 to 4 residual degrees of freedom cannot exclude an interaction of practical size. The feed conductivity imbalance was examined through covariate adjustment. With the initial conductivity as covariate, the microfiltration estimate was +3.07 points (95% CI −3.00 to +9.14, *p* = 0.233) on 4 residual degrees of freedom. The window mean covariate gave +3.34 points (95% CI −1.04 to +7.71, *p* = 0.102), again on 4 residual degrees of freedom. Carrying the block and the initial conductivity together reduced the estimate to +0.72 points (95% CI −3.73 to +5.17, *p* = 0.644) on 3 residual degrees of freedom. In that fit, run order rose to +2.82 points (*p* = 0.048). The conductivity coefficient itself is poorly determined at −0.75 and −0.78 per mS cm^−1^ in the unblocked fits (*p* = 0.581 and *p* = 0.288). Its sign reverses to +0.91 per mS cm^−1^ (*p* = 0.367) once the block enters, a change that suggests instability rather than a genuine reversal. Microfiltration and initial feed conductivity are correlated at r = 0.73, and the variance inflation factors in the joint model are 5.64 for the covariate and 4.02 for the pretreatment term. Adjustment therefore describes the direction of the 1.42 mS cm^−1^ imbalance but cannot separate the pretreatment from the initial conductivity of its feed in this design. Only a new experiment on a homogenized feed appears able to resolve the two. After Holm adjustment across the six reported tests, the smallest *p* value is 0.199 and the microfiltration effect becomes 0.238 (0.286 under Bonferroni). None of the effects reaches the familywise 0.05 level.

The block also removes an apparent difference in run to run agreement. Before blocking, the two runs of the unpretreated trains differ by 3.7 and 3.6 percentage points against 1.0 and 0.7 for the MF-pretreated ones, which invites the reading that pretreatment makes performance more repeatable. After the run order shift is removed, the residual scatter is 0.82 with MF and 0.82 without it. The apparent repeatability advantage is an artefact of run order and not attributed to pretreatment here.

R¯ is the arithmetic mean of the 13 point rejections of a run, each computed from Equation (1) on the feed and permeate conductivities of the same sampling time; R_0_ and R_360_ are its first and last values, EC_p,360_ and pH_p,360_ the permeate conductivity and pH at 360 min, J¯_60–240_ the time-averaged flux over the comparison window, and J_240_ the flux at 240 min. EC_p,vw_ is the volume-weighted mean permeate conductivity of the run, each 30 min reading weighted by the permeate volume collected in its interval (30 to 360 min for the first runs of Plans 3 and 4, whose flux record begins later). The final feed conductivity and pH, the concentration factor, and the recovery bound of each run are given in Table 2.

Estimates are in percentage points of conductivity rejection with 95% confidence intervals. MF and pH are the main effects of the two factors, MF × pH their interaction, and B the run order block, the second run of a cell minus the first. The pooled estimator takes the within-cell variances of the four cells as its error term; the other four fits are least squares regressions of the run mean rejection on the indicator variables. In the full factorial fits, the MF and pH coefficients are simple effects at the reference level of the other factor. The complete model outputs are deposited as Supplementary Materials.

## 4. Discussion

The evidence for the two interventions is asymmetric, and the asymmetry is easily overstated. Microfiltration pretreatment was associated with RO conductivity rejection increased by 2.0 percentage points once run order was blocked, a difference resolved from zero but bounded loosely, from 0.03 to 3.97 points. The feed pH contrast was 0.4 points in the direction the mechanism does not favor and is bounded within ±2 points of zero. What the data do not support is the direct comparison: the contrast between the two interventions is not resolved (*p* = 0.077). The defensible conclusion is therefore about each intervention separately. The pretreatment contrast is one this design could resolve from zero, the acid dosing contrast is one it can bound to be small, and the evidence does not say that pretreatment is the larger of the two. Both statements rest on two runs per cell and on a block that absorbs more than one difference. The feed drawn for the run, the membrane coupon lot, and any drift in the rig are carried together in the run order term and cannot be separated from one another. Nor can the higher initial conductivity of the MF-pretreated feeds be separated from the pretreatment, and the record does not identify its source (Section 2.3). Spread between replicates is visible in every cell, which is why the effects are reported as estimates with confidence intervals rather than as significance decisions and why the power of any single test is low.

The separation between the two membranes follows the expected trade-off. NF270 sustained roughly twice the flux of XLE but rejected only 76 to 80% of the feed conductivity, leaving a permeate of 1.85 to 1.98 mS cm^−1^ at the end of the runs. XLE delivered 0.20 to 0.60 mS cm^−1^ at the same point at 93 to 98% rejection. This ordering matches the general observation that NF membranes offer higher flux and lower salt rejection than RO, quantified for NF270 by Elazhar et al. [11]. The direct comparison of Abu Qdais and Moussa [14] gives the same ordering, with 99.4% removal for RO against 97.4% for NF on the same feed. Srivastava et al. [34] reach the same ordering on brackish groundwater and resolve it by combining the two membranes rather than choosing between them. Where the two are combined, the deciding criterion shifts from rejection to fouling propensity; Hagglund et al. [35] screened a reverse osmosis pilot on a blend of nanofiltration concentrate and brackish groundwater and judged it primarily on fouling behavior. The NF270 rejections observed here were 76 to 80% on a real feed of 4.8 to 8.1 mS cm^−1^. Pak et al. [23] report 22 to 23% in dead-end tests at 70 to 80% recovery and 60% in cross-flow for the same membrane on a synthetic drainage water of about 2.9 mS cm^−1^. Part of the gap follows from the operating conditions. Those tests ran at 15 bar towards high recovery without antiscalant, and the cross-flow run lost more than half of its flux to inorganic scaling within 1.5 h. The present runs, in contrast, recycled the concentrate at 8 bar and reached concentration factors of only 1.6 to 2.7. Whether the NF270 permeate could be applied to a given crop is not a question these data can settle. Suitability depends on the soil and irrigation management as well as salinity, and none of that was tested here. The separation between the two membranes is nonetheless consistent with the view that NF works better as a partial desalination or pre-RO step than as a standalone process for saline feeds [12]. The RO permeate obtained here is comparable to the 375 mg L^−1^ product reported for a lagoon plus two-stage RO train treating 3000 mg L^−1^ Egyptian drainage water [2]. It was achieved at an operating pressure at the lower end of the 6 to 30 bar brackish RO range [12]. The comparison rests on bulk salinity alone. Feed and permeate were characterized by conductivity and pH only, so rejection cannot be reported ion by ion apart from two grab sulfate pairs. The removal of nitrate, the second reason drainage water in this region needs treatment, is not demonstrated here either. The raw water carries 2.6 to 44.7 mg L^−1^ of it as nitrogen across the drainage network that supplied this water.

That the difference with MF appeared on RO but not on NF is consistent with the different roles fouling plays on the two membranes. Agenson and Urase [15] found that organic fouling cost a tight RO membrane 60% of its flux and drove its chloride rejection down from 98.1% to 73.7%. A loose NF membrane under the same treatment lost only 26% of its flux and slightly increased its chloride rejection. That divergence is consistent with narrowed effective pores enhancing size exclusion in a loose membrane even as they impair a tight one, although the authors demonstrate that mechanism for organic solutes rather than for salts. Mahlangu et al. [36] name the mechanism in their review of high-pressure membrane fouling. A deposit raises the concentration at the membrane face, and this cake-enhanced concentration polarization can shift salt rejection in opposite directions depending on the membrane. The two accounts together leave open the possibility that fouling that costs a tight membrane its selectivity leaves a loose one apparently unharmed. The present results follow the same pattern in a much milder form. RO rejection was two points higher with MF pretreatment in both pH branches, while NF rejection was, if anything, marginally lower with MF than without it (77.8 against 78.8%). That is the direction a cake-enhanced contribution to rejection would predict for a loose membrane. That reading is not the only one available for a membrane as loose as NF270. Mahlangu and Mamba [37] fouled NF270 and the tight NF90 with alginate and three colloids under matched hydrodynamics. On NF270, the NaCl rejection fell by 10 to 30% instead of rising, in step with the declining flux as the solution diffusion model predicts. The polarization factor they fitted for alginate fouling was 2.2 on NF270 against 10.4 on NF90, and they concluded that this polarization may not be predominant in membranes that reject salts poorly. The NF trains were not part of the factorial and comprise only two runs each, so the one percentage point separating the two conditions is an observation rather than a test that could decide between these mechanisms. Only one of the two runs on feed as received declined steeply, from 49 to 27 L m^−2^ h^−1^, while its companion run was almost stable, so the loss is recorded as a qualitative observation rather than a result. Flux comparisons between trains also carry an uncorrected term, as the chiller was set to 20 °C but the feed temperature was not logged, so the fluxes reported here are not temperature-normalized. Which pretreatment is chosen also matters, not merely whether one is used. Li et al. [38] set coagulation with sand filtration against ultrafiltration ahead of seawater reverse osmosis and found the two routes to leave different amounts of dissolved organic matter for the membrane to deal with. Cai et al. [39] found that RO membranes fed by differently pretreated secondary effluent fouled to different degrees. Pretreatment is credited with exactly this kind of protection in the literature. Capillary membrane pretreatment of municipal effluent reduced the eventual flux decline of an RO element from 85% to 60% by delivering feed water with far fewer colloidal and suspended particles [17]. Independent evidence of the fouling propensity of this water is available on a longer time scale. In an anoxic membrane bioreactor operated on the same drainage system, the interval between chemical cleanings of a microfiltration membrane was 5 days without gas scouring. It rose to 18 days when the headspace gas was circulated through a venturi to scour the membrane. It fell to 1 to 2 days when the same gas was circulated by a peristaltic pump instead [10]. These figures indicate that fouling on this water is appreciable and that the method of control matters, neither of which can be established from a 6 h batch. Earlier work of the same group on synthetic and municipal wastewater points in the same direction. Venturi-driven scouring extended the time a submerged microfiltration membrane could run before reaching its transmembrane pressure limit by a factor of about three to five relative to a blower at the same aeration rate [40,41]. No membrane autopsy, foulant analysis, or cleaning recovery test accompanies these runs, so the mechanism is inferred from the flux and rejection trajectories alone. The NF runs on drainage water and lost no more flux than the pure water baselines did, which limits how much fouling can be invoked for the NF trains at all.

That no difference with acidification was detected by this design, and none larger than about two percentage points, is the expected result for a feed of this salinity, and the mechanism is well-established. The influence of feed pH on the salt rejection of a polyamide membrane acts through the charge of the active layer and hence through Donnan exclusion of co-ions. Wang et al. [42] measured salt partitioning into the active layers of five polyamide membranes, XLE among them, and found that the Donnan theory predicted the rejection change caused by a pH change to within 0.16 percentage points. The pH response is therefore a charge effect and not a change in solute diffusion. The same work bears directly on a feed of this salinity. It reports that “smaller differences in partitioning between pH 5.3 and 10.5 were observed at greater salt concentration in bulk solution”. At higher ionic strength, the fixed charges of the active layer are screened and the increment in ion exclusion shrinks. Boussouga et al. [43] observed the same screening on NF270, the membrane used here, where the strong pH dependence of arsenate rejection weakened at 20 g L^−1^ NaCl. Our feed ran at 4.8 to 8.1 mS cm^−1^ and concentrated further during each run. That is exactly the regime in which the charge term is screened, and a shift from pH 8 to pH 7 has little left to act on. That account is drawn from the cited studies rather than from these runs. The present measurements are of bulk conductivity on a feed whose ionic composition was not determined. They establish that the pH shift left rejection unchanged, but they cannot demonstrate the screening mechanism offered here as the reason for it. Acid dosing also adds chloride, increasing the monovalent load that a polyamide membrane rejects least effectively, which works against any small gain.

The project report from which these runs originate reached a different conclusion [28]. Acidification left mean removal unchanged there as well at 93.8 and 93.4%, but the flux was read as more sustainable under pH adjustment. That reading rests on one pair of runs. In the pretreated pair, the report records the opposite direction and attributes the lower flux of the acidified train to a feed conductivity of 10.59 against 7.11 mS cm^−1^. The interpretations differ in what they attribute the flux difference to. The pH-adjusted runs were performed on the more saline feeds of the study. Once the flux is expressed against net driving pressure, the pH effect is not resolved in any plausible parameterization of the osmotic correction (Section 3.5, Figure 5). A statistical adjustment cannot separate the two terms. Feed salinity is aliased with the pH factor in this design, so entering conductivity as a covariate leaves both terms unidentifiable. The separation rests on the physical correction, not on a regression, and what it supports is the absence of a demonstrated flux effect rather than proof that acid dosing is inert. The absence of a demonstrated flux effect does not rule out the benefit for which acid dosing is normally sought in practice. That benefit was not the target here: the pH arm asks whether removal changes when the naturally alkaline feed is brought to neutral. Acidification targets carbonate scale, as the solubility of calcium carbonate increases as pH decreases, as noted by Thompson et al. [5]. That scale develops over far longer operation and at far higher recovery than these 6 h runs reached. Recovery itself cannot be quoted directly because the feed tank volume was not recorded and the concentration factors reached here bound it only loosely (Section 2.4). Pilot work on real wastewater over 610 h reports acid only as a cleaning agent applied once fouling had developed, not as feed conditioning [44].

Even over scaling-relevant operation, however, acidification would address only part of the risk on water of this type. Thompson et al. [5] reported that the solubilities of gypsum, strontium sulfate, and barite are comparatively insensitive to pH, so antiscalant dosing rather than acidification has been the usual mitigation for such salts. Agricultural drainage waters commonly favor this sulfate pathway. According to Tang and Liu [4], their calcium ranges from 200 to 600 mg L^−1^ and their sulfate from 2000 to 20,000 mg L^−1^, and gypsum precipitation can then induce severe membrane scaling. In agreement with that assessment, McCool et al. [3] indicated that achievable recovery on brackish drainage water may be limited because such waters are often near or above saturation with respect to gypsum, calcium carbonate, and silica. The feed examined here contained 1050 and 1250 mg L^−1^ sulfate in two grab samples, so a sulfate-driven scaling risk appears plausible. The calcium, magnesium, and bicarbonate concentrations that would set the scaling potential were never measured, however, so no saturation index can be computed, and the premise of the intervention cannot be evaluated even retrospectively. Against this background, a rejection response to a modest pH shift bounded within two percentage points of zero accords with what the literature would suggest. McCool et al. [3] found that for all but one of the drainage water sources evaluated, operation at the natural alkaline pH of approximately 7.5 to 8 produced a lower degree of membrane scaling than acidic operation. Competitive crystallization effects outweighed the reduction in calcite supersaturation that thermodynamic solubility analysis alone would predict. The pH arm accordingly neither supports nor contradicts [3], whose endpoint was scale formation rather than rejection. This design can test whether feed pH changes rejection, and feed pH does not. It cannot test whether feed pH protects flux over a scaling-relevant time scale, and the literature gives no reason to expect an obvious gain from acidification on water of this composition.

The concentration factors attained over the eight RO runs bound recovery between roughly 25 and 54%, largely below the range at which field systems on comparable waters have operated. McCool et al. [3] estimated an expected product water recovery of 44 to 68% across the San Joaquin Valley. The field assessment of Thompson et al. [5] sustained an operational recovery of 58 to 65% on drainage water of 14,440 mg L^−1^ TDS, with permeate salinity in the range of 370 to 400 mg L^−1^. That pilot used a membrane of the same family as the elements studied here and reached 97.7% observed rejection at 18.7 bar and 63% recovery. The present runs operated at 8 bar on a much less saline feed in batch concentration mode at bench scale and without antiscalant. The field values therefore serve to locate the bench conditions rather than to benchmark performance against them. Because most runs stayed below the recovery window in which those systems operate, scaling behavior at field-relevant recovery was never reached, and a configuration recommendation cannot be drawn from these experiments.

A further observation concerns the behavior of rejection under concentration. In the NF runs, the feed conductivity rose by a factor of 1.6 to 2.7, yet observed rejection increased from 72 to 80% at the start of the runs to 79 to 85% at 360 min. Rejection rising with feed concentration has been reported for low-pressure membranes over a wide concentration range [21]. Concentration polarization theory offers a compatible reading, in that flux falls as feed concentration rises in the polarized regime, which reduces the convective solute flux towards the membrane relative to the back-diffusive flux [45]. Because the initial feed conductivity also differs between replicates, this trend should be read as a within-run observation, not a controlled concentration series.

On the feeds treated here, microfiltration pretreatment was associated with a gain in rejection under a post hoc run order block, and the single loss of membrane permeability occurred in an unpretreated run. These observations describe what happened on this rig at 8 bar and do not amount to a recommendation for any treatment train. Recommending a configuration would require water recovery, energy consumption, chemical use, cleaning frequency, membrane area, and brine management data, none of which this campaign produced. Over the 6 h acute cycles studied, feed pH adjustment to approximately 7 was accompanied by no measurable difference in rejection. Acid dosing in practice is applied as a scaling control measure, however, and this design cannot address scaling; calcium, magnesium, and bicarbonate, the ions that set scaling potential, were not measured on any sample. The two membranes used here and the operating principles they illustrate remain in current use, but these results should be read as a study of process configuration rather than any specific membrane product.

Future work on this water should resolve rejection by ions, including nitrate, chloride, and the divalent cations that govern scaling risk. It should extend the runs at a controlled recovery so that the concentration trajectory is set instead of observed. It should also compare microfiltration pretreatment against antiscalant dosing, which addresses a different fouling mechanism than the one microfiltration appears to control here. Nitrate behavior cannot be read from the conductivity data. Zeggar et al. [46] fitted irreversible thermodynamics transport models to nitrate transport on an NF and an RO membrane using synthetic nitrate solutions of 50 to 150 mg L^−1^ and a nitrate-rich brackish groundwater. The nanofiltration membrane proved markedly sensitive to the initial nitrate concentration, while the reverse osmosis membrane performed stably, with a solute permeability roughly an order of magnitude lower. Nitrogen removal lay outside of the scope of this design, but it has been demonstrated on the same water through hydrogenotrophic denitrification in an anoxic membrane bioreactor [10]. A train that combines biological nitrogen removal with membrane desalination therefore merits evaluation for water in which both the nutrient and the salt load limit reuse.

## 5. Conclusions

At a documented 8 bar operating point on real Harran Plain drainage water, low-energy brackish water RO (XLE) rejected 93 to 98% of feed conductivity, whereas loose NF (NF270) rejected 76 to 80%. This separation is the firmest result of the study and indicates which membrane class could serve reuse on this water, subject to the qualifications below. Of the two interventions examined, microfiltration pretreatment was associated with higher rejection in these runs at 2.0 percentage points (95% CI 0.03 to 3.97 points). That estimate rests on a block on run order chosen after the pattern was seen, and no result withstands familywise adjustment. Feed pH adjustment produced no comparable difference, its effect being bounded within two percentage points of zero, although the two interventions are not resolved from each other. The single loss of membrane permeability occurred in an unpretreated run; with one loss in eight runs, that observation cannot carry weight on its own. Several features of the design bound what can be concluded. The two runs of each train drew on a storage tank that was replenished during the series and started at different conductivities. Water recovery was not measured and is only bounded by the concentration factor, and irrigation suitability was not tested; conductivity alone does not establish it. Until recovery is measured on this water, the transfer of these rejections to field operation remains untested. The data describe acute rejection and flux behaviour over 6 h batch cycles on these feeds; they cannot support a recommended treatment train, statements about scaling control, or estimates of cost or energy demand. With two runs per condition and characterisation limited to conductivity, this is a preliminary, descriptive case study of process configuration on one drainage water rather than a study of membrane-level mechanisms. The next step for this water is ion-resolved testing at controlled recovery on a homogenized feed split across the treatment conditions in more than two blocks so that matched feeds and adequate replication are part of the design.

## Figures and Tables

**Figure 1:**
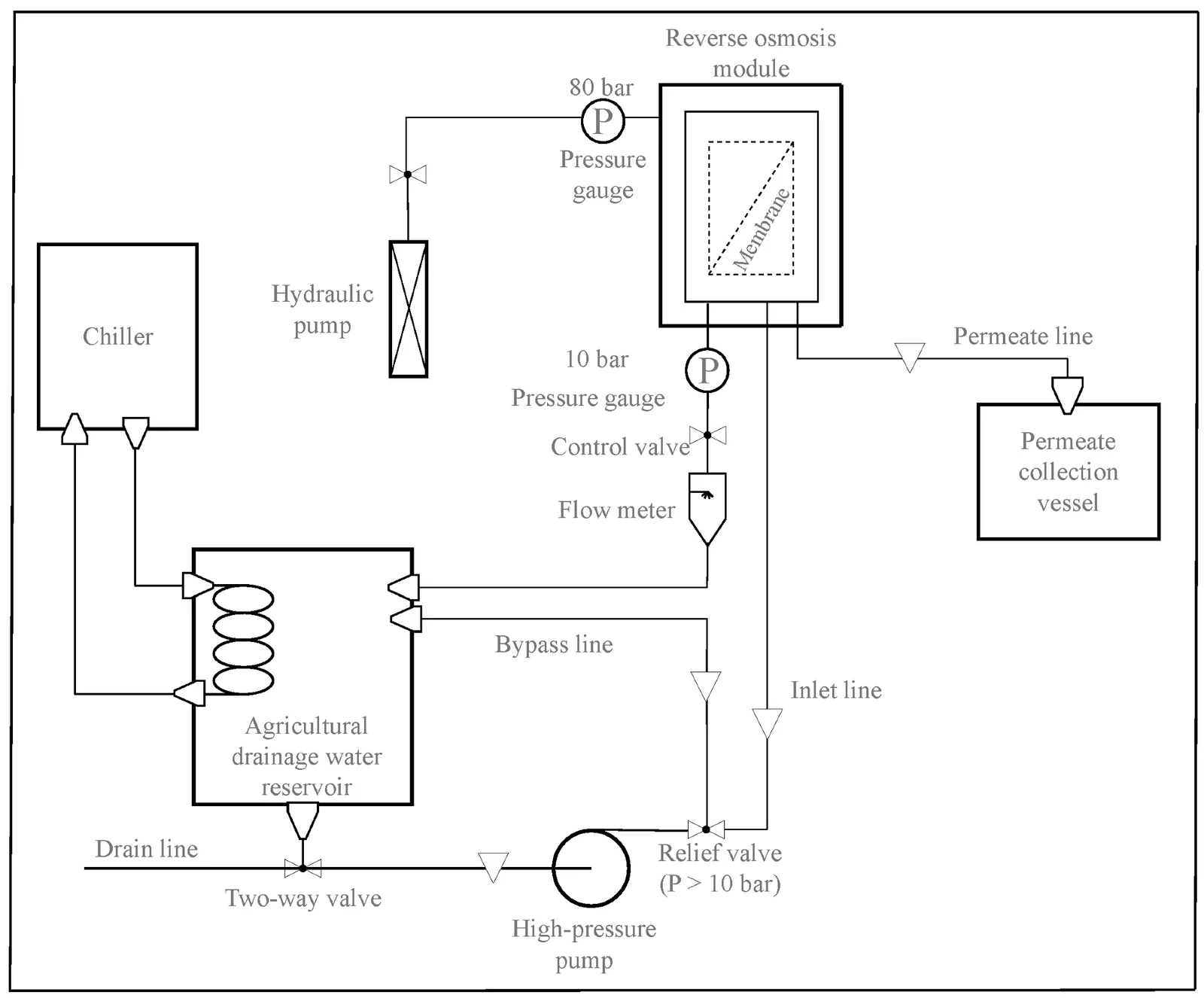
Process flow diagram of the laboratory cross-flow membrane system.

**Figure 2:**
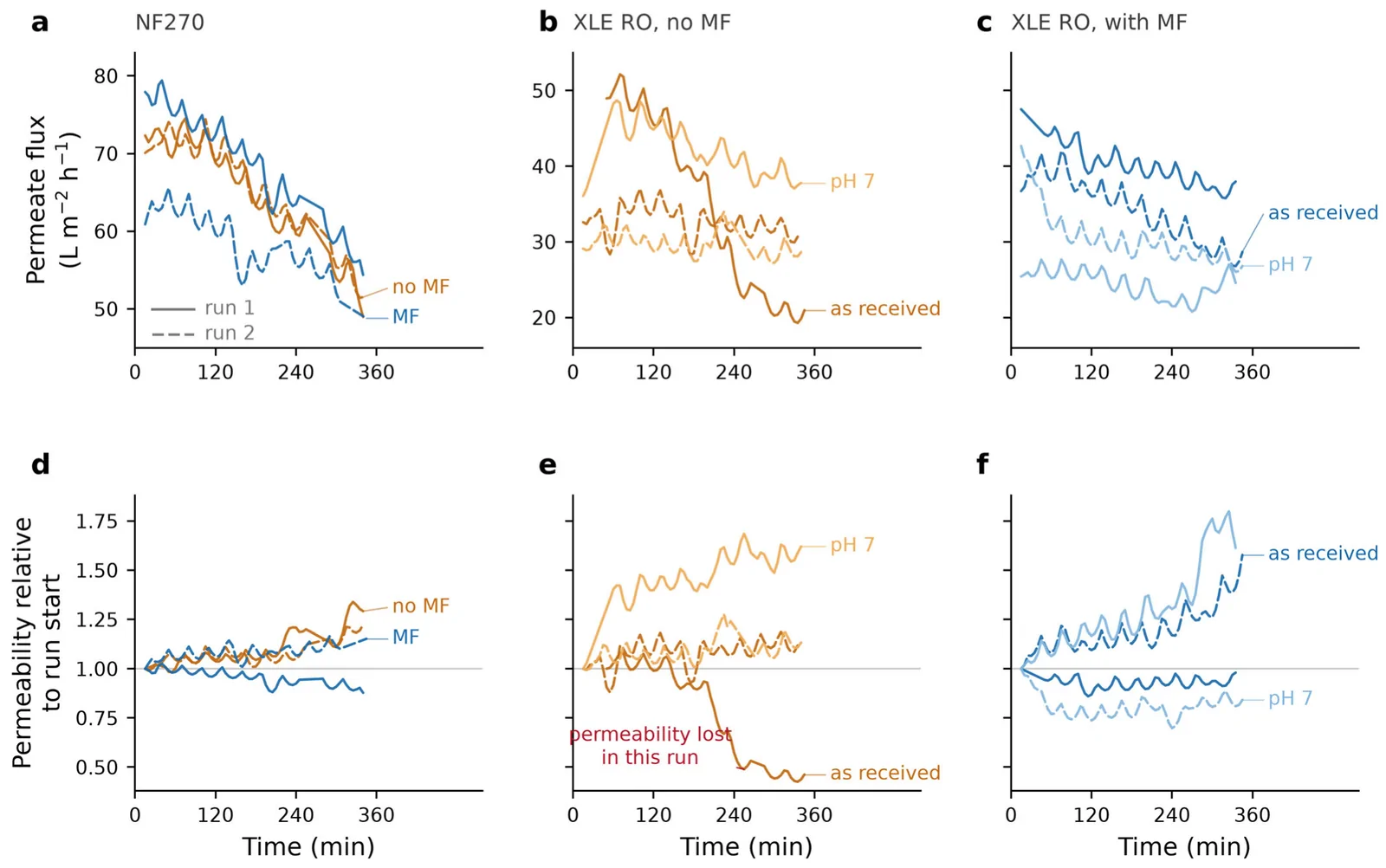
Permeate flux and net-driving-pressure-normalized permeability of the NF and RO trains: (**a**) permeate flux of the two NF270 trains; (**b**) flux of the XLE trains on feed as received; (**c**) flux of the XLE trains with MF pretreatment; (**d**–**f**) the corresponding runs expressed as permeability relative to the start of the run (Section 2.5), in the same column order.

**Figure 3:**
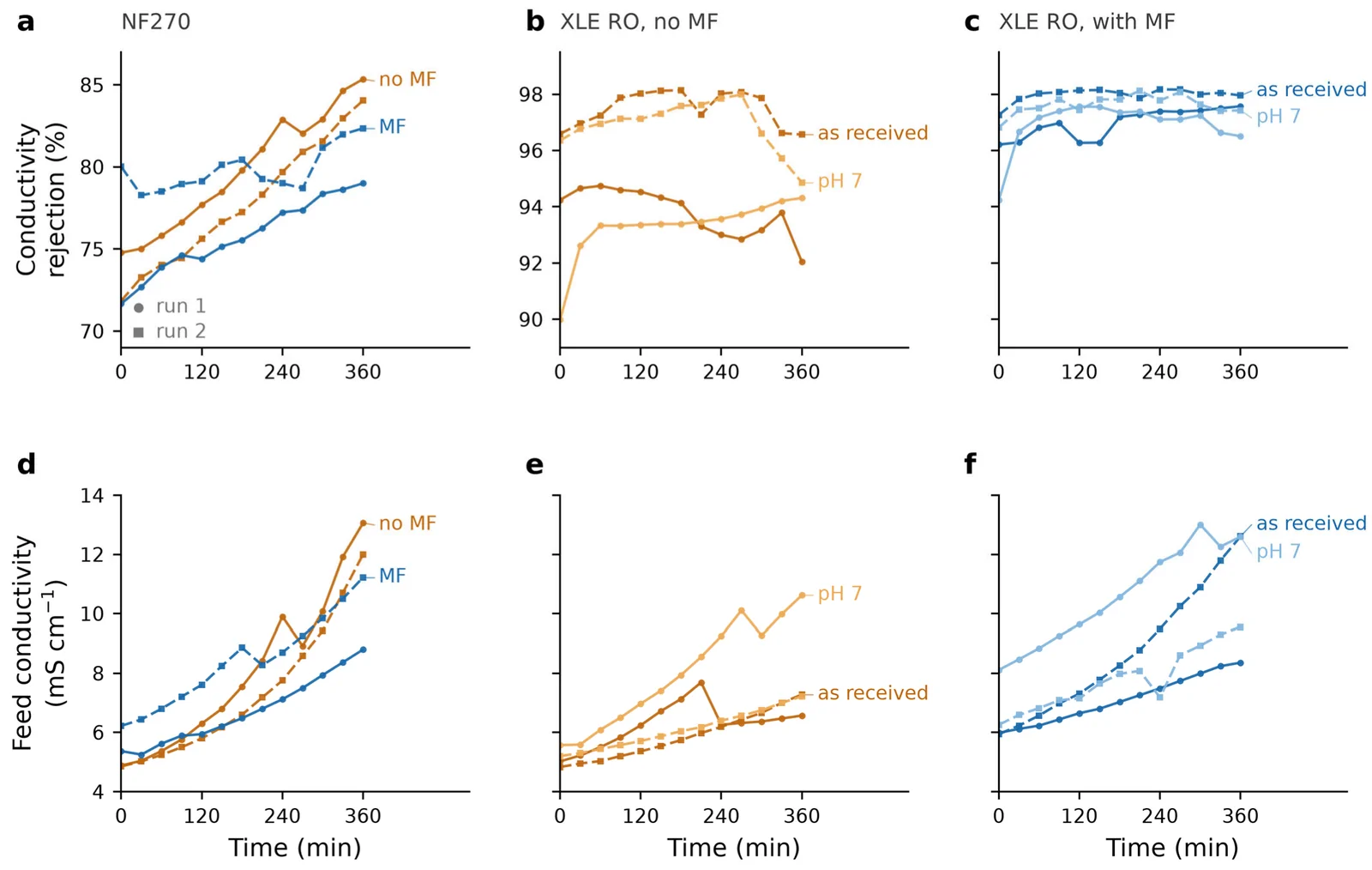
Conductivity rejection and feed conductivity during the batch concentration runs: (**a**) conductivity rejection of the two NF270 trains; (**b**) rejection of the XLE trains on feed as received; (**c**) rejection of the XLE trains with MF pretreatment; (**d**–**f**) feed conductivity of the corresponding runs, in the same column order.

**Figure 4:**
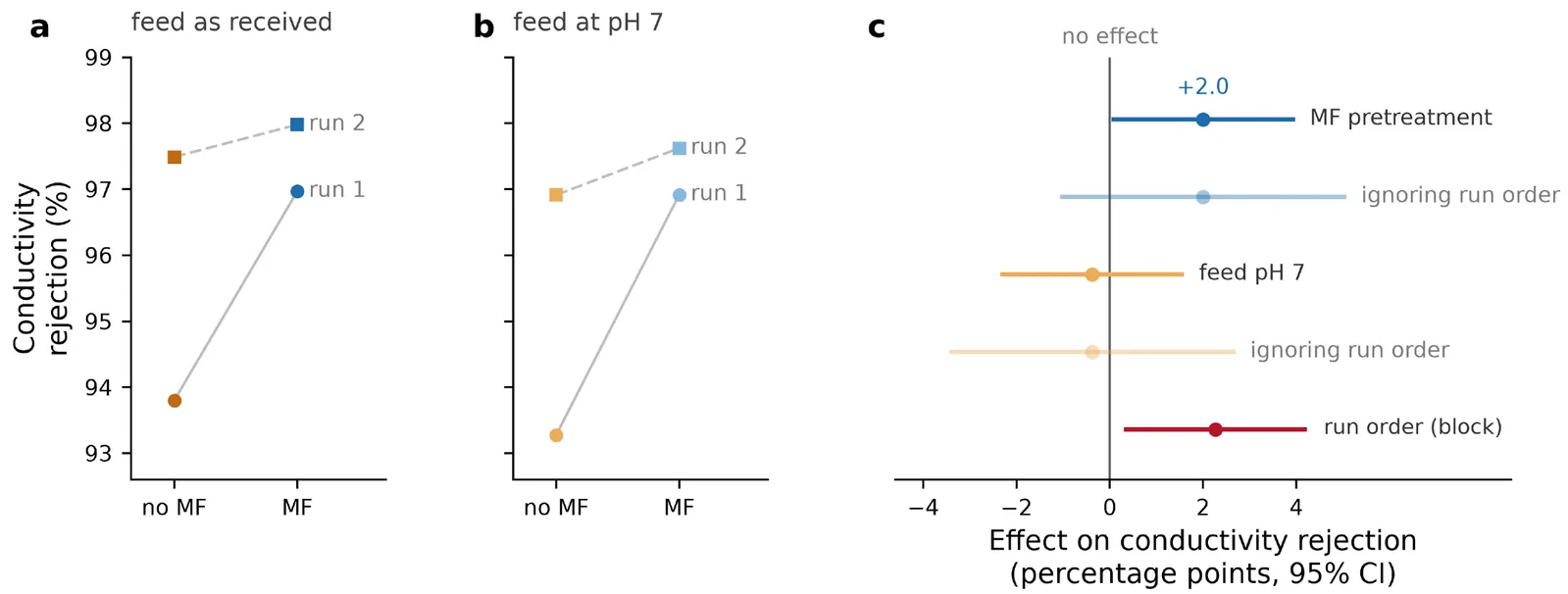
Run-level rejection and the estimated effects of pretreatment, pH adjustment, and run order: (**a**) mean conductivity rejection of the XLE runs against MF pretreatment for the feed as received; (**b**) the same for the feed at pH 7; (**c**) estimated effects of MF pretreatment, feed pH 7, and run order on rejection with 95% confidence intervals, shown with the run order block and, in the paler tone, without it.

**Figure 5:**
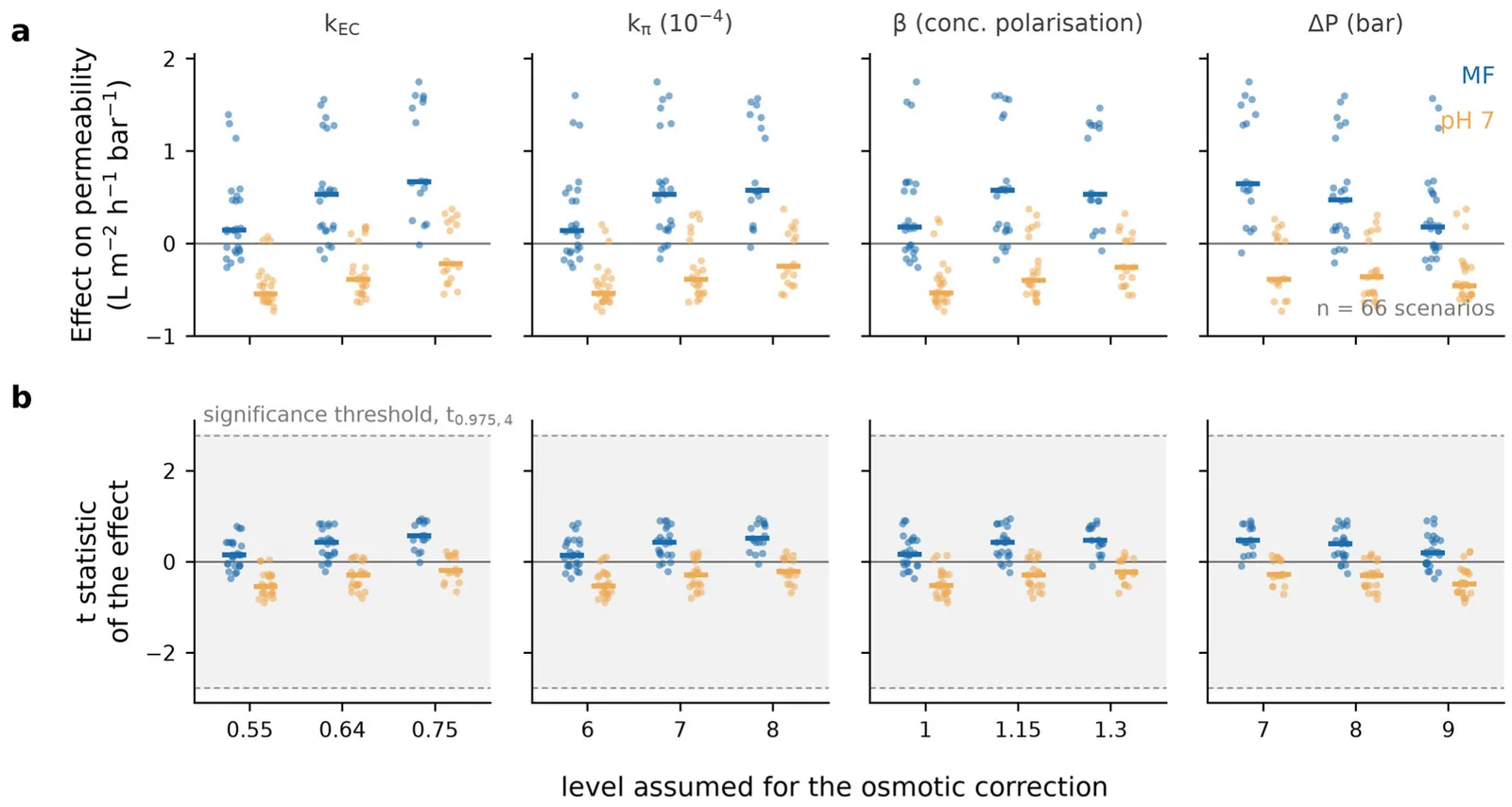
Sensitivity of the estimated factor effects to the assumptions of the osmotic correction: (**a**) effect of MF pretreatment and of feed pH 7 on permeability in each admissible scenario, grouped by the level assumed for each of the four coefficients; (**b**) the corresponding t statistics against the significance threshold.

**Table 1:** Treatment trains.

Plan	Train	Membrane	MF	pH Adjustment
1	ADW + CART + NF	NF270	no	no
2	ADW + CART + MF + NF	NF270	yes	no
3	ADW + CART + RO	XLE	no	no
4	ADW + CART + MF + RO	XLE	yes	no
5	ADW + CART + pH + RO	XLE	no	yes
6	ADW + CART + MF + pH + RO	XLE	yes	yes

ADW, agricultural drainage water; CART, coarse cartridge filter; MF, microfiltration; NF, nanofiltration; RO, reverse osmosis. Each train was run twice (Section 2.3).

**Table 2:** Run metadata and feed conditions of each run.

Plan	Rep	Train	EC_f,0_ (mS cm^−1^)	EC_f,360_ (mS cm^−1^)	pH_f,0_	pH_f,360_	CF (EC_f,360_/EC_f,0_)	Recovery Bound (%)
Plan 1	1	CART + NF	4.88	13.06	8.21	8.51	2.7	n.a.
	2	CART + NF	4.85	12.00	8.16	8.41	2.5	n.a.
Plan 2	1	CART + MF + NF	5.37	8.80	8.08	8.61	1.6	n.a.
	2	CART + MF + NF	6.22	11.22	8.33	8.76	1.8	n.a.
Plan 3	1	CART + RO	5.02	6.57	7.96	8.59	1.3	25
	2	CART + RO	4.83	7.28	7.90	8.33	1.5	34
Plan 4	1	CART + MF + RO	5.99	8.36	8.21	8.72	1.4	29
	2	CART + MF + RO	5.94	12.61	7.96	8.65	2.1	54
Plan 5	1	CART + pH + RO	5.57	10.62	7.02	7.32	1.9	50
	2	CART + pH + RO	5.20	7.22	7.07	7.04	1.4	29
Plan 6	1	CART + MF + pH + RO	8.11	12.60	7.06	6.95	1.6	36
	2	CART + MF + pH + RO	6.26	9.56	7.09	6.89	1.5	35

**Table 3:** Per-run performance summary of the twelve drainage water runs.

Plan	Rep	Mean Rejection, R¯ (%)	Rejection at 0 min, R_0_ (%)	Rejection at 360 min, R_360_ (%)	Permeate EC at 360 min, EC_p,360_ (mS cm^−1^)	Volume-Weighted Permeate EC, EC_p,vw_ (mS cm^−1^)	Permeate pH at 360 min, pH_p,360_	Mean Flux 60 to 240 min, J¯_60–240_ (L m^−2^ h^−1^)	Flux at 240 min, J_240_ (L m^−2^ h^−1^)
Plan 1	1	79.8	74.8	85.3	1.91	1.48	8.69	67.0	59.6
Plan 1	2	77.7	71.8	84.0	1.92	1.50	8.50	67.6	60.1
Plan 2	1	75.8	71.7	79.0	1.85	1.57	8.67	70.1	63.5
Plan 2	2	79.8	80.0	82.3	1.98	1.66	8.80	58.9	55.6
Plan 3	1	93.8	94.2	92.0	0.52	0.39	8.19	41.9	26.5
Plan 3	2	97.5	96.6	96.6	0.25	0.13	7.65	33.3	31.7
Plan 4	1	97.0	96.2	97.6	0.20	0.21	8.56	40.9	39.1
Plan 4	2	98.0	97.3	98.0	0.26	0.16	8.45	35.9	30.8
Plan 5	1	93.3	90.0	94.3	0.60	0.52	7.14	44.1	39.8
Plan 5	2	96.9	96.3	94.9	0.37	0.18	6.75	30.1	32.8
Plan 6	1	96.9	94.2	96.5	0.44	0.30	6.83	24.6	21.5
Plan 6	2	97.6	96.8	97.4	0.25	0.18	6.65	30.2	27.8

**Table 4:** Coefficients of the five fits of mean conductivity rejection over the RO factorial.

Fit	Residual d.f.	Term	Estimate (Points)	95% CI	*p*
Pooled 2 × 2	4	MF	+2.00	−1.70 to +5.70	0.207
	4	pH	−0.38	−4.07 to +3.32	0.792
	4	MF × pH	+0.17	−3.52 to +3.87	0.903
Additive, unblocked	5	MF	+2.00	−1.07 to +5.07	0.154
	5	pH	−0.38	−3.44 to +2.69	0.766
Full factorial, unblocked	4	MF	+1.83	−3.40 to +7.06	0.387
	4	pH	−0.55	−5.78 to +4.68	0.785
	4	MF × pH	+0.35	−7.05 to +7.74	0.903
Additive, blocked	4	B	+2.26	+0.29 to +4.23	0.033
	4	MF	+2.00	+0.03 to +3.97	0.048
	4	pH	−0.38	−2.34 to +1.59	0.624
Full factorial, blocked	3	B	+2.26	−0.32 to +4.84	0.069
	3	MF	+1.83	−1.83 to +5.48	0.210
	3	pH	−0.55	−4.20 to +3.11	0.665
	3	MF × pH	+0.35	−4.82 to +5.51	0.845

## Data Availability

The raw laboratory workbook, the reproducible analysis notebook, the derived time series, the summary tables, and the complete model outputs are provided as Supplementary Materials. The notebook regenerates every derived file from the workbook. The remaining records of the campaign form part of the archive of TÜBİTAK project 114Y453 and are available from the corresponding author upon request.

## Supplementary Materials

The following supporting information can be downloaded at: https://www.mdpi.com/article/10.3390/membranes16100310/s1, Supplementary File S1 (HarranRO_Supplementary.zip): a README describing the package; the raw laboratory workbook; the analysis notebook; the derived time series and per-run summaries; the complete model outputs, including the osmotic-correction sensitivity grid and the flux processing audit; and Tables S2 and S3 as editable files.
