# Supplementary material for: Effects of Microfiltration Pretreatment and Feed pH Adjustment on Nanofiltration and Reverse Osmosis Desalination of Agricultural Drainage Water
Source: Membranes (Basel). 2026 Sep 23;16(10):310. doi: 10.3390/membranes16100310 (PMC13706926; doi:10.3390/membranes16100310)
Supplement: Supplementary file 1 [file membranes-16-00310-s001.zip › HarranRO_Supplementary/README.pdf]

Supplementary Materials

Effects of microfiltration pretreatment and feed pH adjustment on nanofiltration and reverse osmosis desalination of agricultural drainage water.

Contents

Path	What it holds
data/Deney Seti.xlsx	The raw laboratory workbook: 25 sheets holding the balance logs and the pH/EC series of every run.
HarranRO_pipeline.ipynb	The analysis end to end, from the raw workbook to every reported number. Outputs are cleared; running it writes the same files into outputs/.
derived/	Time series and per-run summaries built from the raw records.
models/	Every model fit reported in the paper, with coefficients, intervals, degrees of freedom and the sensitivity sweep.
tables/	Table S2 and Table S3 as written into the manuscript.

Derived series

File	Content
ec_timeseries.csv	Feed and permeate conductivity and pH, 13 points per run
flux_timeseries.csv	Permeate flux from the balance log
ro_runs_ndp.csv	Per-run flux, net driving pressure and permeability
rejection_summary.csv	Per-run rejection, concentration factor, endpoints
flux_summary.csv	Point and window-mean fluxes per run

Model outputs

File	Content
factorial_effects.csv	Pooled 2^2 estimator: main effects and interaction for R, J and A
factorial_blocked.csv	Additive fits with and without the run-order block
factorial_crosscheck.csv	Raw flux and rejection regressions, no

File	Content
	osmotic model
revision_model_table.csv	Four model specifications side by side
revision_ec_covariate.csv	Feed conductivity entered as a covariate
revision_multiplicity.csv	Holm and Bonferroni adjustment of the reported tests
ph_equivalence_tost.csv	TOST for the pH effect
revision_tost_90ci.csv	The 90% interval TOST corresponds to, and the margins
sensitivity_osmotic.csv	81 osmotic-correction scenarios, 66 admissible
revision_recovery_bounds.csv	Recovery bounded from the concentration factor
revision_purewater_permeability.csv	Clean-water permeability and drift envelope
revision_ph_trajectory.csv	Within-run feed pH trajectory
revision_table3_additions.csv	End-of-run feed and permeate values
revision_flux_processing_audit.csv	Segment boundaries and excluded tails of every balance log
revision_tail_threshold.csv	Window-mean flux at three flat-tail thresholds
revision_matched_cf.csv	Rejection at a matched concentration factor, volume-weighted permeate EC

Sheet key of the raw workbook

Sheet names are in Turkish, as recorded during the campaign.

Sheet	Content
Deney Planı	The experimental plan
NF safsu, NF safsu (2)	NF270 pure-water baselines
RO safsu, RO safsu (2), RO safsu (3)	XLE pure-water baselines
Plan 1 to Plan 6, and each (2)	The twelve drainage-water runs, two per train
Run 1-2(Tüm Akılar), NF(Tüm Akılar), RO(Tüm Akılar)	Flux series collected together
RO(pH etkisi), MF+RO(pH etkisi)	pH comparisons assembled by the original author
NF(MF etkisi), RO(MF etkisi)	Pretreatment comparisons, likewise

Each run sheet holds two blocks: columns A to D are the 15 s balance log and columns F to K the 30 min pH and conductivity series. The analysis uses the run and baseline sheets

and recomputes everything from them; the last seven sheets are the original author's own comparisons and are not read by the notebook.

Running it

Python 3.12 with pandas, numpy, scipy, statsmodels, matplotlib and openpyxl. Run the notebook from the directory that holds it; it reads data/Deney Seti.xlsx and writes its results into outputs/. The files under derived/, models/ and tables/ are those same outputs, grouped, so every number in the paper can also be checked without running anything.
