# Supplementary material for: Effects of Microfiltration Pretreatment and Feed pH Adjustment on Nanofiltration and Reverse Osmosis Desalination of Agricultural Drainage Water
Source: Membranes (Basel). 2026 Sep 23;16(10):310. doi: 10.3390/membranes16100310 (PMC13706926; doi:10.3390/membranes16100310)
Supplement: Supplementary file 1 [file membranes-16-00310-s001.zip › HarranRO_Supplementary/tables/tableS2_feed.pdf]

Table S2. Initial feed properties and concentration factor of each run.

Plan	Rep	Train	EC_f,0 (mS cm⁻¹)	pH_f,0	CF (EC_f,360/EC_f,0)
Plan 1	1	CART+NF	4.88	8.21	2.7
Plan 1	2	CART+NF	4.85	8.16	2.5
Plan 2	1	CART+MF+NF	5.37	8.08	1.6
Plan 2	2	CART+MF+NF	6.22	8.33	1.8
Plan 3	1	CART+RO	5.02	7.96	1.3
Plan 3	2	CART+RO	4.83	7.90	1.5
Plan 4	1	CART+MF+RO	5.99	8.21	1.4
Plan 4	2	CART+MF+RO	5.94	7.96	2.1
Plan 5	1	CART+pH+RO	5.57	7.02	1.9
Plan 5	2	CART+pH+RO	5.20	7.07	1.4
Plan 6	1	CART+MF+pH+RO	8.11	7.06	1.6
Plan 6	2	CART+MF+pH+RO	6.26	7.09	1.5
