# Supplementary material for: Effects of Microfiltration Pretreatment and Feed pH Adjustment on Nanofiltration and Reverse Osmosis Desalination of Agricultural Drainage Water
Source: Membranes (Basel). 2026 Sep 23;16(10):310. doi: 10.3390/membranes16100310 (PMC13706926; doi:10.3390/membranes16100310)
Supplement: Supplementary file 1 [file membranes-16-00310-s001.zip › HarranRO_Supplementary/tables/tableS3_summary.pdf]

Table S3. Per-run performance summary (rejection %, permeate EC, point fluxes in L m^{-2} h^{-1}).

Plan	Rep	$\bar{R}$	R_0	R_{360}	EC_p,360 (mS cm^{-1})	$\bar{J}_{60-240}$	J_{240}
Plan 1	1	79.8	74.8	85.3	1.91	67.0	59.6
Plan 1	2	77.7	71.8	84.0	1.92	67.6	60.1
Plan 2	1	75.8	71.7	79.0	1.85	70.1	63.5
Plan 2	2	79.8	80.0	82.3	1.98	58.9	55.6
Plan 3	1	93.8	94.2	92.0	0.52	41.9	26.5
Plan 3	2	97.5	96.6	96.6	0.25	33.3	31.7
Plan 4	1	97.0	96.2	97.6	0.20	40.9	39.1
Plan 4	2	98.0	97.3	98.0	0.26	35.9	30.8
Plan 5	1	93.3	90.0	94.3	0.60	44.1	39.8
Plan 5	2	96.9	96.3	94.9	0.37	30.1	32.8
Plan 6	1	96.9	94.2	96.5	0.44	24.6	21.5
Plan 6	2	97.6	96.8	97.4	0.25	30.2	27.8
